# A unique self-organization of bacterial sub-communities creates iridescence in *Cellulophaga lytica* colony biofilms

**DOI:** 10.1038/srep19906

**Published:** 2016-01-28

**Authors:** Betty Kientz, Stephen Luke, Peter Vukusic, Renaud Péteri, Cyrille Beaudry, Tristan Renault, David Simon, Tâm Mignot, Eric Rosenfeld

**Affiliations:** 1 UMR 7266 CNRS- Littoral Environnement et Sociétés, Microbial Physiology Group - Université de La Rochelle, Avenue Michel Crépeau, 17042 La Rochelle, France; 2 School of Physics, University of Exeter, Exeter EX4 4QL, United Kingdom; 3 Laboratoire Mathématiques, Image et Applications EA 3165, Université de La Rochelle, France; 4 Institut Français pour la Recherche et l’Exploitation de la Mer, Unité Santé Génétique et Microbiologie des Mollusques, Laboratoire de Génétique et de Pathologie des Mollusques Marins, La Tremblade, France; 5 UMR 7283 CNRS Laboratoire de Chimie Bactérienne, Institut de Microbiologie de la Méditerranée, University of Aix-Marseille, Marseille, France

## Abstract

Iridescent color appearances are widespread in nature. They arise from the interaction of light with micron- and submicron-sized physical structures spatially arranged with periodic geometry and are usually associated with bright angle-dependent hues. Iridescence has been reported for many animals and marine organisms. However, iridescence has not been well studied in bacteria. Recently, we reported a brilliant “pointillistic” iridescence in colony biofilms of marine *Flavobacteria* that exhibit gliding motility. The mechanism of their iridescence is unknown. Here, using a multi-disciplinary approach, we show that the cause of iridescence is a unique periodicity of the cell population in the colony biofilm. Cells are arranged together to form hexagonal photonic crystals. Our model highlights a novel pattern of self-organization in a bacterial biofilm. ”Pointillistic” bacterial iridescence can be considered a new light-dependent phenomenon for the field of microbiology.

The use of light and color is important in biology. For example, living organisms have developed original colorations. Pigmentation, bioluminescence and fluorescence are types of coloration that have been largely documented, especially in marine organisms^1^,^2^,^3^. They share a biochemical nature. Conversely, iridescence is defined as a structural color. It is due to the interaction of light with micron- and submicron-sized physical structures spatially arranged with periodic geometry (e.g. photonic crystals^4^). These structures can interact with incident light to create preferential scattering of specific spectral colors which can generate the most conspicuous colour appearances and highly functional optical effects often associated with communication. Angle-dependence is a defining characteristic of iridescence^4^,^5^,^6^. Iridescent color appearances are widespread in nature. The various animals (notably insects and birds) exhibiting structural colors have been the subject of keen interest to both the biology and physics fields. Iridescence has also been reported in marine animals (fishes, crabs, mollusks and jellyfishes^7^,^8^,^9^,^10^,^11^,^12^,^13^), macroalgae^14^,^15^, diatoms^16^,^17^ and in one virus system^18^.

In contrast, prokaryotic iridescence has been ambiguously described or overlooked in the literature. In bacteria, iridescence was not observed at the individual-scale (*i.e*. the cell) but was only visualized in bacterial colonies or concentrated cells suspensions^19^ (and references therein). In early works, rainbow-diffuse iridescences^20^,^21^,^22^,^23^,^24^,^25^,^26^,^27^(observed under trans-illumination) were reported notably in strains of *Haemophilus influenza*, however, their underlying mechanisms were not described. In addition to this, metallic iridescence of some *Pseudomonas aeruginosa* isolates and mutant strains was found to be correlated to the accumulation of 4-hydroxy-2-alkylquinoline (HAQ) molecules^28^,^29^,^30^. Again, the mechanism was not unveiled.

Recently, we have isolated an intensely iridescent bacterium, *Cellulophaga lytica* CECT 8139, from a sea anemone surface in the Charente Maritime coast (France)^19^. Iridescence of the colony biofilms is observed under epi-illumination under different growth conditions (see Fig. 1 and Supplementary Movies S1,S2,S3,S4,S5 for typical examples). *C. lytica*’s iridescence is intense and easily visualized under natural light exposure contrary to previously reported rainbow-diffuse bacterial iridescences. The term “glitter-like” iridescence was previously used, but since similar pixelated structural colors have been recently described as “pointillist”^31^, *C. lytica*’s iridescence was here renamed “pointillistic” iridescence. This discovery raises key questions about the possible function, if any, of its specific iridescent appearance.

Although bacterial iridescence is yet to be observed in the natural environment (e.g. as iridescent epibionts of marine organisms), the microbiological relevance of this new phenotype was reinforced by recent works. We found that iridescence occurred under a wide range of culture conditions notably under psychrophilic, halophilic and hydric stress conditions that mimic the natural biotope of *C. lytica*, *i.e*. rocky shore ecosystem^19^,^32^. Marine organisms have already been reported as iridescent in such ecosystem^14^,^15^. Optical characterisation (using spectrophotometry) and structural analyses (using electron microscopy) confirmed the structural coloration and to show the color-coded intensity of scattered light as a function of wavelength and observation direction.

Strongly reflected wavelengths spanned near-ultraviolet, visible and near-infrared; in view of this potential biological roles such as thermoregulation, dessication prevention or photoprotection^4^ should be investigated further. Similar iridescence was elicited in several other *Cellulophaga* spp. and Bacteroidetes originating from culture collections^33^ or from the French Atlantic coast^34^. Many iridescent bacteria were isolated from seaweeds or marine invertebrates as epibionts. For all iridescent strains, a strong link between gliding motility and the establishment, in time and space, of the iridescent appearance was evidenced^33^,^34^. However, the nature of the necessary periodic structures responsible for this unique pointillistic iridescence remained completely unknown.

Here, we aimed at identifying the mechanistic cause of *C. lytica*’s iridescence. By using a multi-field approach (microbiology, microscopy and image processing techniques, physical modeling), we propose and describe the first model of physical explanation for iridescent color appearance in the prokaryotic kingdom. We show that sub-communities of bacterial cells can form remarkable highly ordered spatial organizations to create iridescence.

## Results and Discussion

### Statistics of color and luminance profiles of iridescent *Cellulophaga lytica* colonies

**Figure 2:**
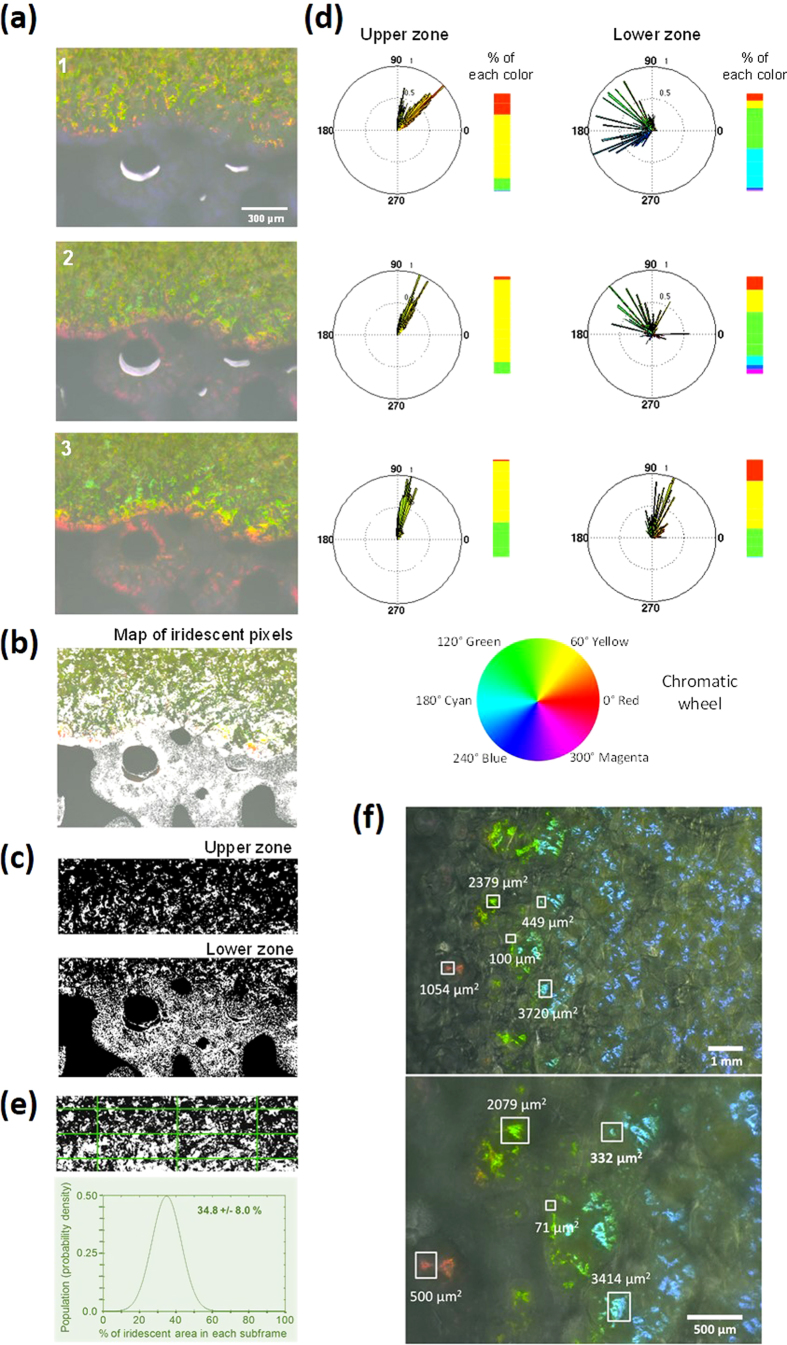
Color variations at the edges of an iridescent *C. lytica* CECT 8139’s colony grown on marine agar (MA). (**a**) Optical digital microscopy images (× 200) were taken at high (1), intermediate (2) and low (3) light incidence angles. (**b**) Processed image showing the iridescent pixels (superimposed in white to the original image) varying in color or luminance between images (1) to (2) and/or (1) to (3). (**c**) Separate processing of the upper and lower zones showing the iridescent pixels varying in color or luminance at the three incidence angles. In that case, iridescent pixels that appear or disappear in at least one image were not recorded. (**d**) Color profiles of the iridescent pixels (from (**c**)). Statistics were obtained by computing circular histograms on the Hue channel. Luminances were normalized from 0 to 1 (with 0.5 and 1 diameter values being shown on the circular histograms). As illustrated by the chromatic wheel, each color corresponds to an angle interval: red [0:30°], orange [30:45°], yellow [45:75°], green [75:120°], cyan [~180°], blue and magenta [~240–300°]. Percentages of each color were calculated to give a simplified view of the color profiles. (**e**) Distribution analysis of pointillist iridescent regions in the upper zone. The areas of iridescent pixels were computed within a regular grid. The resulting frequency plot is shown. (**f**) Statistic determination of “speckle” sizes within a *C. lytica*’s colony. Optical digital microscopy images were taken at × 100 (top image) and × 200 (image below) magnifications. In this example (a CYT-grown colony), a zone with several speckle colors was analyzed.

The visible appearance of the colonies of the marine bacterium *Cellulophaga lytica* CECT 8139 was recently described but its mechanism was not elucidated^19^. To this end, we have analyzed the iridescence signatures of large zones of many *C. lytica* colonies at three illumination angles (low, intermediate and high incidences). Figure 2(a–d) shows examples of the results obtained at the edges of a marine agar (MA)-grown colony. Colonies’ spectral reflectance signatures were strongly dependent on illumination angles, notably at the peripheral edges of the colony (Fig. 2c,d). A great variety of colors (yellow, green, cyan, blue, magenta, red, orange) could be observed.

We measured the areas of the iridescent structures that varied in color and/or luminance between 2 and 3 illumination angles (low, intermediate and high incidences). Figure 2(b) and Supplementary Dataset S1 summarize the results obtained with the same MA-grown *C. lytica* colony, the identified iridescent pixels appearing in white. In the upper zone of the image, the total iridescent area represented 44% of the total area while in the lower zone, the iridescent area reached more than 84% of the colonized area. This indicates that iridescent color appearance can arise in large area-proportions of *C. lytica* colonies. Similar analyses were repeated for other colonies and growth conditions (Supplementary Dataset S2). Results are summarized in Supplementary Dataset S3.

To study the distribution of iridescent regions further, we divided the image of the upper zone with a regular grid and computed the area and standard deviation of iridescent pixels for this zone (example shown in Fig. 2(e)). The iridescent area for each subframe was 34.8%, with a standard deviation of 8.0% confirming the visual observation of a nearly spatially homogeneous distribution of pointillist iridescent areas. Similar results, with even smaller standard deviations, were obtained for many other *C. lytica* colonies (data not shown). Iridescent centres are clearly abundant over the surface of *C. lytica* colonies.

### The iridescent elemental units, the “speckles”, are similar in size to sub-communities of bacterial cells

In order to determine the dimensions of the physical structures responsible for iridescence, it was crucial to determine the dimensions of the iridescent elemental units, the iridescent “speckles”. In order to take into account the potential heterogeneity in size, we favored the colony edges in which diverse cell densities were present and in which diverse colors were observed. In the example shown (Fig. 2(f)), five speckles (blue, green and red) were analyzed computationally. A large heterogeneity in size was found (see also Supplementary Dataset 4) with the smaller iridescent elemental units occupying approximately 50 μm^2^ (Fig. 2(f) shows a 71 μm^2^ speckle). This minimal iridescent elemental unit size, suggests the color centres are formed by bacterial cell sub-communities, namely clusters of (motile) cells.

### TEM images of iridescent colonies show remarkable organizations of bacterial sub-communities

**Figure 3:**
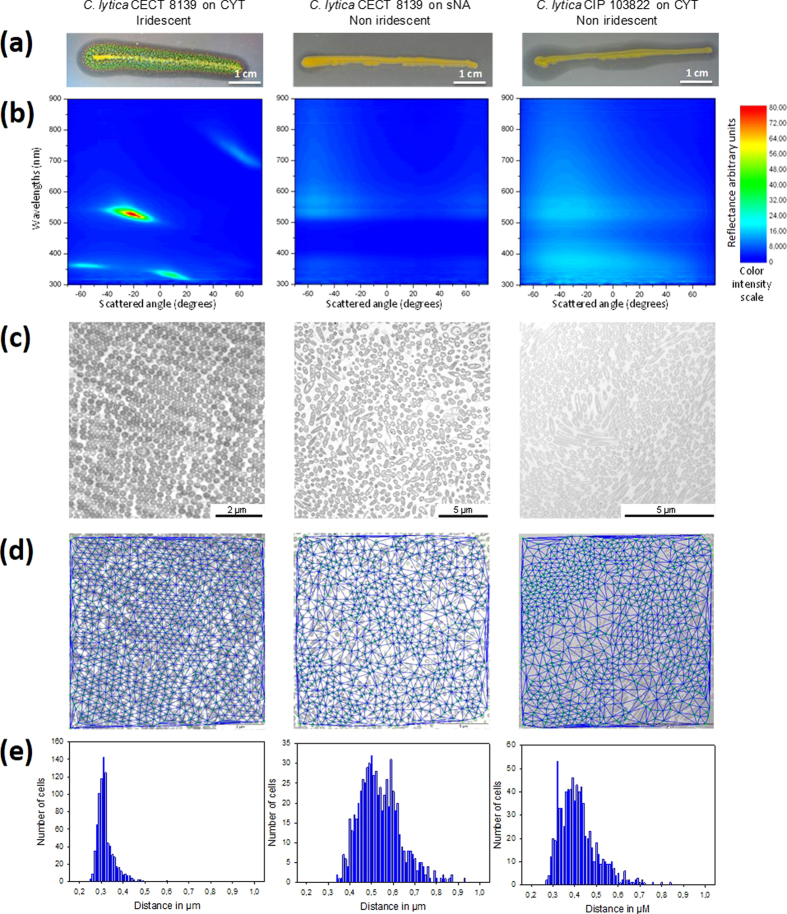
Spectrophotometry of iridescent and non-iridescent *C. lytica* colonies and TEM and Delaunay triangulations of the internal structures. (**a**) Appearances of the colonies (streaking method on agar plate). (**b**) For spectrophotometry of assays, samples were illuminated at a fixed light angle of −70°. Scattered wavelengths from 300 nm to 850 nm were recorded at different detection angles from −65° to 70° with 2° angle step resolution. Results are shown using a color intensity scale for each scattered wavelength. Wavelength values for each color were as follows: UV, <400 nm; violet, 400 to 435 nm; blue, 435 to 490 nm; cyan, 490 to 520 nm; green, 520 to 560 nm; yellow, 560 to 590 nm; orange, 590 to 620 nm; red, 620 to 700 nm; and infrared, >700 nm. (**c**) Transmission Electron Microscopy (TEM) images of colony cross-sections obtained by using an adapted protocol (see *Methods*). (**d**) Extraction of periodic structures from TEM images with Delaunay triangulations. (**e**) Each histogram represents the frequency plot of the mean distance from the six nearest neighbours for all the cells in the image. Values are the associated mean distances from the six nearest neighbours and standard deviations.

Spectrophotometry was used to quantify physically the iridescence of cytophaga (CYT)-grown *C. lytica* CECT 8139 colonies^19^ and compare them to non-iridescent sNA-grown *C. lytica* CECT 8139 and CYT-grown *C. lytica* CIP 103822 colonies (Fig. 3(a,b)). The color map of CYT-grown *C. lytica* CECT 8139 colonies shows optical reflection bands that unequivocally represent the iridescence by the change in reflected color with angle. This was shown for distinct zones of *C. lytica*’s colonies grown on MA, CYT and low nutrient (LN) media (data not shown). For non-iridescent colonies, only a very low optical reflection, probably caused by the yellow pigment zeaxanthin^35^, was recorded. All of these growth conditions were used as positive or negative controls to elucidate the underlying mechanisms responsible for iridescence.

**Figure 4:**
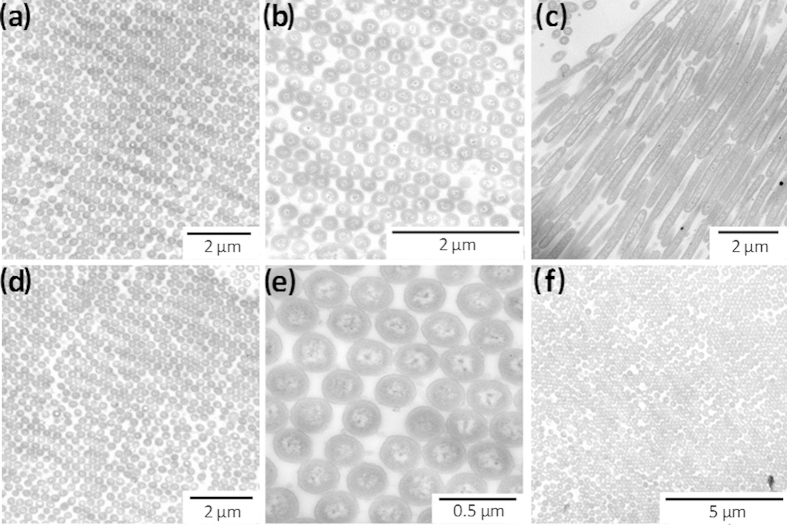
TEM cross-section images of *C. lytica* CECT 8139 colonies under different iridescent conditions. **(a–e)** *C. lytica* was aerobically grown for 24 h at 25 °C on Cytophaga agar (CYT). **(f)** *C. lytica* was aerobically grown for 24 h at 25 °C on Marine Agar (MA).

TEM images of colony cross-sections revealed a remarkable organization of bacterial cell sub-communities for conditions leading to iridescent reflection (Fig. 4). The bacterial cells had the same orientation, appeared equally spaced and aligned on the entire image (~50 μm^2^). Visualization of the cells in their longitudinal orientation indicated they were very close to each other on their entire edges. For all conditions that gave rise to iridescence a high degree of organization of cells in the sub-communities was visible even where sharp changes of orientation occurred (Supplementary Dataset S5). Under conditions that did not give rise to iridescence, the structural organization within sub-communities of cells appeared low and even absent in CYT-grown CIP 103822 and sNA-grown CECT 8139 colonies, respectively (Fig. 3(c), Supplementary Dataset S5). These data suggest that the cell organization within sub-sections of the bacterial population is the cause of iridescence. Even if small groups of organized cells were visible in CYT-grown CIP 103822, the number of arranged cells may not be sufficient to create discernable iridescence.

The same strains and conditions were examined by using phase contrast microscopy. Gliding motility, collective motions and a high degree of cell organization were visible only under conditions that give rise to iridescence (see Supplementary Movies S6 to S10).

### Mathematical morphology and Delaunay triangulation demonstrate clearly the observed periodicity

TEM images of the iridescent colonies revealed quasi-ordered structural geometry. The Delaunay triangulation algorithm applied to these TEMs on cells centers enabled the quantification of cells’ spatial distributions (Fig. 3(d,e)) and statistical data describing cell dimensions. Under iridescent control conditions, the distribution of measured mean distance of every cell to its six nearest neighbours (6NN) was extremely narrow (322 nm ± 38 nm). Much broader distributions of cells’ 6NN distances (535 ± 96 nm, 416 ± 87 nm) were recorded for non-iridescent control conditions. Additional statistical data are shown in Supplementary Dataset S6.

### Elucidation of *C. lytica*’s iridescence using physical modeling

**Figure 5:**
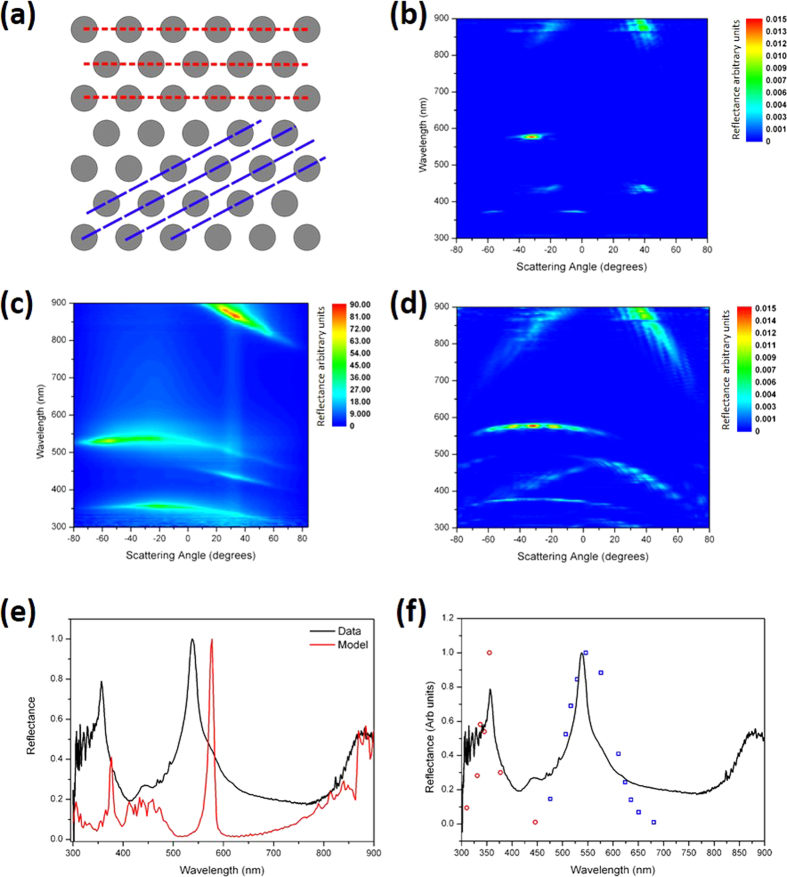
Comparison of experimental and modeled light scattering data and reflectance spectra. **(a)** Schematic diagram of the model geometry. Two sets of scattering planes have been highlighted. **(b)** Modeled scattering pattern for a hexagonal-packed array with a periodicity of 320 nm. Data are shown (as in (**c,d**) using a color intensity scale for each scattered wavelength. **(c)** Experimental light scattering data. The sample was illuminated at an incident angle of −30°. **(d)** Modeled scattering pattern which comprises a weighted average of scattering patterns from many models with a range of geometric orientations. **(e,f)** Experimental and modeled reflectance spectra, calculated by summing optical scatter in the reflectance hemisphere. **(e)** Experimental (black line) and modeled (red line) reflectance data, calculated by summing the optical scatter plotted in **(a)** (experimental) and **(d)** (modeled). **(f)** Experimental reflectance data (as in **(e)**) overlaid with modeled reflectance, calculated using a multi-periodicity approach and a weighted average derived from the periodicity displayed in Fig. 3(d,e) and Supplementary Dataset S6.

Finite element method modeling (http://www.uk.comsol.com) was used to verify the optical phenomena responsible for the “pointillistic” iridescence of *C. lytica* and a modeling protocol was devised to mimic the experimental conditions. TEM images indicated that the bacterial cells form colonies comprising spatial domains of close-packed structure. Image analysis of many TEM sections yielded a mean cell spatial periodicity of 322 ± 38 nm (Fig. 3(e)). The identified close-packed colony structures actually appear in large domains, rather than as a continuous uniform single structure. Between these domains the orientation of the close-packed structure varies. This domaining was incorporated into the model using an averaging technique. The intra-domain geometry was rotated from −30° to + 30° degrees between models with the 0° orientation comprising a close-packed structure with horizontal planes of bacteria, as shown in Fig. 5(a). A weighted average of the scattering patterns produced from each domain orientation was calculated.

If a single close-packed model with a periodicity of 320 nm and with horizontal planes of bacteria (see Fig. 5(a)) is considered, the scattering pattern appears as several localised areas of high reflectance such as in Fig. 5(b). These reflectance maxima (e.g. at 450 nm, 580 nm and 885 nm) approximately correspond to specular Bragg reflections from the planes of bacteria schematically shown in Fig. 5(a). Such modeled data does not match all aspects of the reflectance observed experimentally (Fig. 5(c)), for instance its angular range. This can be attributed to the domained or polycrystalline nature of the cell colonies. TEM images confirm the orientation of the planes within each domain of periodic order changing from one colony to the next. This plays a significant role in determining the scattering angle at which the reflectance maxima are observed. Experimentally, many polycrystalline domains were illuminated by the incident beam spot. The measured response (Fig. 5(c)) therefore, is a spatial average of the reflectance from many domains. A distribution of planar orientations within the beam spot results in the angular broadening of the reflectance maxima. To model this, the colony structure within our model was rotated in 5° steps up to ± 30°. In each case, a strong correlation was found between the expected and the observed scattering angle at which reflectance maxima were recorded. A concurrent change in reflected wavelength accompanied the change in scattering angle. A Gaussian distribution of planar orientations was assumed, with its centre occurring for a geometry rotation of 0° (*i.e*. representing cell planes parallel to the sample surface). This Gaussian profile was used as a weighting function in the calculation of the mean scattering pattern. This modeled scattering pattern is shown in Fig. 5(d). With this approach the modeled reflectance maxima that appear in Fig. 5(b) become extended over a wider scattered angle range and the scattering pattern becomes a much closer match to that seen experimentally.

It should be noted, however, that the modeled reflectance maxima are very narrowband when compared to the experimental data. This is apparent when the scatter plots (Fig. 5(c,d)) are summed over all scattering angles. These spectra, both experimental and modeled, are plotted in Fig. 5(e). The modeled reflectance spectrum has a similar number of maxima; they are, however, red-shifted and very narrow-band when compared to the experimental data. Along with geometrical orientation, there is evidence of a distribution of intra-domain periodicities; this distribution is plotted in Fig. 3(e). As the experimental beam-spot incorporates many local structural domains of close-packed bacilli the observed series of macroscopic reflectance spectra are convolutions of the many individual spectra that result from differences in the structural periodicity and orientation of different individual domains. This effect broadens the reflected wave-band into a continuous spectral feature.

The periodicity distribution plotted in Fig. 3(e) was used to determine a range of model periodicities. Ten periodicities from 260 nm to 375 nm were modeled and each scattering pattern generated was summed to give an associated reflectance spectrum. The dominant reflectance maximum from each of the ten models, weighted by a function derived from the periodicity distribution plotted in Fig. 3(e), are plotted in Fig. 5(f) along with the experimental reflectance spectrum. An improved agreement between experiment and model is now evident; the modeled reflectance maxima are now aligned with the experimental data and are considerably broader-band.

This modeling indicates that a range of inter-domain periodicities and geometry orientations are responsible for the broadband and broad-angle reflectance maxima observed in the experimental scattering patterns.

In conclusion, these results provide the first physical explanation of bacterial iridescence. The mechanistic cause of iridescence is a unique periodicity and quasi-order of bacterial cell sub-communities within the colony biofilm. The manner in which “pointillistic” iridescence is created in bacteria, and especially in marine *Flavobacteriaceae* capable of gliding motility^33^, can now be studied in detail as a result of having available the appropriate tools in microbiology (strains and culture conditions), optics (microscopy techniques), mathematics (image processing algorithms applied to pointillistic iridescence or TEM images) and physics (modeling protocol). This iridescence phenomenon, which was generally overlooked in prokaryotes, is potentially widespread^32^,^33^,^34^ and might be used as a new taxonomic criterion for the field of microbiology. However, bacterial iridescence is yet to be observed in the natural (marine) environment.

A strong link was found between gliding motility of the cells and the establishment, in time and space, of the iridescent structures. The iridescent structures (groups of cells) themselves can move^33^. The (inter)cellular mechanisms, the genes and metabolites involved in the onset of iridescence, as well as their biological roles^4^,^19^,^32^,^33^,^34^ are unknown for now.

Independently from iridescence, the present study highlights a unique pattern for bacterial cells in a colony biofilm. This opens up exciting perspectives in the field of microbiology, notably to help with the understanding of the multicellular organization within bacterial biofilms. Surprisingly, a similar pattern was very recently observed in rotating spherical cells of the Proteobacterium *Thiovulum majus*, but this phenomenon occurs in planktonic conditions, in a drop of water on a microscope slide^36^.

## Methods

### Bacterial strains and culture conditions

Culture media comprised ready-to-use Marine agar (MA) medium from Dutscher (Laboratorios Conda, S.A. Pronadisa^®^) and salted Nutrient agar (sNA) (Dutscher) with a supplement of NaCl 25 g L^−1^. Prepared media were made with artificial seawater (ASW) Instant Ocean© (30 g L^-1^). Cytophaga agar (CYT) contained 1 g tryptone, 0.5 g yeast extract, 0.5 g CaCl_2_.2 H_2_O, 0.5 g MgSO_4_.7 H_2_O, and 15 g agar in 1 L of ASW^37^,^38^. This medium was adapted with casein replaced by tryptone because *C. lytica* does not degrade casein. Low nutrient medium (LN) contained 15 g of agar in 1 L of ASW^39^. For a selected set of samples, the MA medium was supplemented with black ink (Paper Mate^©^ 1% (v/v)) that was sterile-filtered using a 0.2 μ PES membrane filter. This was done to enhance color contrast for some of the optical measurements and led to no discernable detrimental effect on the bacterial growth.

Temperature of incubation was fixed at 25 °C. Iridescence of *Cellulophaga lytica*’s colonies was not influenced by light exposure during incubation^32^.

*C. lytica* CECT 8139 isolated from an anemone surface in the French Charente Maritime coast (France)^19^ was principally employed in this study. Colonies of this strain exhibit “pointillistic” iridescence on several marine media (MA, CYT, LN) but are non-iridescent on the salted medium sNA. Green iridescence with red and violet iridescent colony edges correspond to the profile observed on CYT and MA. A specific green iridescent profile is visible on LN^19^. *C. lytica* CIP 103822 was additionally used as a negative control because its colonies are non-iridescent on all media^19^,^33^. When needed, iridescence of bacterial colonies was compared with the aid of a streaking procedure^19^.

### Observations by optical digital microscopy

Direct observations of colored colonies were performed under epi-illumination by using a Keyence microscope (VHX-1000E). A VHX-1100 camera with a VH-Z20R/Z20W objective lens could be adjusted at ×50, ×100, ×200 magnifications^19^. To avoid specular reflections, the VH-S30 supporting mount of the camera was oriented at a 60° angle from the plate. The DEPTH UP/3D tool corresponding to the depth-from-defocus (DFD) process was employed at high magnification to focus on all optical fields and to improve image quality. These conditions were used for estimating the size of the iridescent speckles (see Statistical Analysis below).

In order to observe transitory color reflections, the VHX-1100 camera was equipped with a VH-K20 lens ring. The support for the camera was mounted at a 90° angle with respect to the plate. By moving the ring from right to left, three positions of illumination were used, namely, high, intermediate, and low light incidence angles. Due to the rapid gliding motility of *C. lytica* CECT8139 cells, the three images were captured rapidly (in a few seconds).

### Time-lapse phase-contrast microscopy

Gliding motility was followed in phase-contrast using an automated and inverted epi-fluorescence microscope TE2000-E-PFS (Nikon, France). The microscope was equipped with “The Perfect Focus System” that automatically maintains focus on a point of interest. Images were recorded with a CoolSNAP HQ 2 (Roper Scientific, Roper Scientific SARL, France) and an oil immersion Nikon 100×/1.4 DLL objective. Due to the short working distance of 100× objective, a 1 cm sided cube of colony growing on agar was cut and inverted on a coverslip.

### Physical measurement of *C. lytica’s* iridescence (spectrophotometry)

Illumination was directed onto the sample through an Ocean Optics UV-Vis-NIR optical fibre that was connected to an Ocean Optics HPX-2000 light source that spans approximately 300 nm to 850 nm. An Ocean Optics 74-ACR collimating lens directed light from the end of the fibre to the sample surface. The reflected light was collected using a similar lens and optical fibre that was itself connected to an Ocean Optics USB4000-UV-VIS spectrometer^19^. The angle of illumination and of detection could be separately set and controlled to a resolution of 0.5°. For a series of chosen fixed illumination angles, the collection fibre was stepped in 2° angle steps in an arc over the sample, and reflection spectra were recorded at each angular position. Reflectance intensity from samples was normalised against an Ocean Optics WS-1 white reflectance standard.

### Preparation of samples for transmission electron microscopy and analysis of images

An adapted protocol was developed in order to preserve the internal structures of the colonies growing at the surface of agar plates during the invasive steps of sample preparation for Transmission Electron Microscopy (TEM). The principle was to enclose the colony into soft agar and to use this agar bubble for further treatments. A piece of a 24 h-growth colony was covered with soft salted agar (consisting of 6 g L^−1^ of agar and 30 g L^−1^ NaCl) cooled at 40 °C. A “bubble” of agar was formed around the colony. Iridescence could still be observed through the soft agar and after gelling. For each condition, three agar-enclosed colonies were treated for TEM fixation, dehydration and contrast. All chemicals were purchased from Sigma.

The protocol used was adapted from already existing ones^40^. The first step of TEM preparation was an overnight fixation bath in glutaraldehyde 3% (in cacodylate buffer) at 4 °C. After two rinse cycles of 10 minutes in cacodylate buffer (10 mL cacodylate 0.4 M, 4 mL NaCl 10% and 6 mL pure water; pH 7.4; 1100 mOsm), fixation and contrast were performed in osmium tetroxide 1% solution (1 mL Osmium tetroxyde 4%, 1 mL cacodylate 0.4 M, 1 mL NaCl 10% and 1 mL pure water) during 1 h at 4 °C. Samples were again rinsed twice with cacodylate buffer during 10 minutes. For dehydration, a graded series of alcohol steps were used (10 min 30%, 10 min 50%, 10 min 70%, 2 × 15 min 95%, 3 × 20 min 100%,) and finally, samples were immersed twice in propylene oxide for 15 min. Impregnation in the resin followed immersion in baths of propylene oxide and resin (v/v) for 1 h and in pure resin for 1 h. The resin was made with 18.48 g Epon, 9.3 g DDSA (Dodecyl succinic anhydre), 9.3 g MNA (Methyl-5-Norbornene-2,3-dicarboxylic Anhydride),0.45 g DMP3O (2,4,6-Tris(dimethyl-aminomethyl)phenol). The agar-enclosed colony was then cut in smaller pieces disposed at the bottom of a 500 μL Eppendorf tube full of resin and left for reticulation at 60 °C for 48 h with an orientation that permitted colony cross-section. Resin blocks were then removed from the plastic tube and cut to form a rectangular plane surface before cutting with an Ultrotome (LEICA EM UC6). Semi-thin sections (1 μm) were stained with toluidine blue and screened for areas with cells. Ultrathin sections were mounted on copper grids, contrasted with uranyl acetate and lead citrate and examined using a JEOL JEM-1011 transmission electron microscope at 60 kV.

### Statistical analysis of pointillistic iridescence variation

All image processing programs used in this study have been developed using the Matlab programming environment. Transitory colors of the colonies were analysed using three microscopic images corresponding to the same colony section for three illumination conditions: high, intermediate, and low light incidence angles.

Iridescent elements in a field of view imaged by a digital camera may be characterized both by highly saturated and bright pixels. To analyze these elements in our images of *C. lytica*, we performed a color space transformation, from the more conventional RGB (Red Green Blue) representation of digital images to HSV space (Hue Saturation Value). After this transformation only pixels having high values in both Saturation and Value channels were considered. These pixels in the image correspond to iridescent areas in the field of view of the sample. Statistics on those iridescent pixels were performed by computing circular histograms on the Hue channel^41^. Briefly, the color of a pixel is represented in the HSV cylindric system, where the Hue is an angle between 0 degree (red) and 360 degrees. In order to study the statistics of the Hue value for iridescent pixels, a circular histogram is computed, which is a polar plot showing the distribution of values grouped according to their numeric range.

For each image, the total percentages of pointillist iridescent areas were calculated, as well as the relative percentage of each color. These values were calculated for each of the three illumination angles. The influence of the illumination angle on the pointillist color reflection was also studied. Depending on the angle, regions of the colony can change in color, luminance or both. Those behaviors were analyzed separately and were represented by variation maps. The program calculates the image areas, or the percentage of those iridescent structures, that vary in color and/or luminance between two illumination angles. A binary version of the image with two colors was then obtained, typically the background color and pointillist iridescent structures.

For computing statistics of color profiles at the extreme edges of *C. lytica* colonies (e.g. lower zone in Fig. 2(c,d)), pixels having (at one, two or three angles) a color too close to the background color were removed.

### Statistical analysis of the dimension of iridescent speckles

The area corresponding to iridescent zones of the colony was extracted from classical optical microscopy at different scales. A program was developed for estimating the size of iridescent structure (speckle) from a selected area in the image. Based on the property of high saturation of potentially iridescent pixels, the area of iridescent structures was estimated in μm^2^.

An average area of pointillist iridescent structures was then calculated, as well as a histogram of their size distribution. Different images at different scales of the same zone enabled confirmation of the consistency of the analysis.

### Mathematical morphology analysis of the TEM images

Statistical analyses of images were needed to confirm the presence of a network and evaluate its order and related dimensions. In our study, the information collected from TEM images comprised: the distances between the centers; the distances between the edges; the alignment of cells and the network formed. The image processing technique used for extracting this information was based on mathematical morphology^42^. This describes mathematical theory and techniques for the analysis of structures. It is a widely used tool in image processing because it can efficiently extract image components that are useful for representing and describing image regions (such as borders, or the notion of convex hulls). The two basic operations of mathematical morphology are dilation and erosion, which correspond to the union (or intersection) of the image with translations of a form called structuring element. In our application framework, mathematical morphology allowed us to extract the individual cells of an image, including their center, their shape and their average size (using granulometric analysis^41^). Once the cell network was extracted, statistical studies of the spatial distribution of these cells could be performed.

All automatic image processing methods for detecting objects in a scene can miss some potential targets. Here, the few missed detections could have been manually corrected, but they will not significantly change the distribution statistics of the cells spatial arrangement.

#### Image segmentation

The extraction of the cell network was performed in several steps. First, a granulometric analysis provides an estimate of the average cell size in the image, allowing for further processing to determine the size of the structuring element to be employed. Morphological filtering was then used to extract the geometry of cells, and a thresholding operation (using Otsu thresholding^43^) was performed to get a binary image (1: cell; 0: background). Cells that were not entirely included in the image were removed in order not to bias the statistics, and cell center coordinates were finally extracted, as well as the area of ​​cells, their structure and their size (function: *regionprops* in Matlab).

#### Extraction of the cells network

Information extracted in the previous step allowed the analysis of the network formed by the cells contained in the image. A Delaunay triangulation was performed with the centers of each cell in order to show more clearly the observed structure. A Hough transform^41^,^44^ was also used to extract the main axes’ alignment.

### TEM image modeling by *Comsol multiphysics*

A modeling protocol, using the commercial modeling package Comsol Multiphysics, was devised to mimic the experimental technique. Due to the cigar-shaped nature and 2D periodicity of the structure, a 2D modeling technique was adopted. Each model comprised a 10 μm wide environment representing the agar medium (refractive index = 1.34^45^) with the bacteria (refractive index = 1.38^46^,^47^,^48^) represented as solid circles in a hexagonally-arranged geometry with a fixed periodicity of between 260 nm and 375 nm. Although some fine structure detailing is visible in the inner part of the TEM images of some bacterial bacilli, their stain-generated grey-scale contrast is not significantly different from the outer part of its structure. For this reason, the refractive index assigned to each bacillus’ unit in our model did not vary with radial distance from its centre. The bacteria in our model were assigned a fixed diameter of 250 nm. Illumination via a plane-wave was incident upon the bacteria geometry with an incident angle of 30 degrees. The reflection hemisphere of the model comprised a semi-circular space, the boundary of which was resolved into 1^°^ segments. The reflectance across each of the 1^°^ segments was calculated; this provided a modeling approach analogous to the experimental method.

## Additional Information

**How to cite this article**: Kientz, B. *et al*. A unique self-organization of bacterial sub-communities creates iridescence in *Cellulophaga lytica* colony biofilms. *Sci. Rep*. **6**, 19906; doi: 10.1038/srep19906 (2016).

## Supplementary Material

Supplementary Information

Supplementary Movie S1

Supplementary Movie S2

Supplementary Movie S3

Supplementary Movie S4

Supplementary Movie S5

Supplementary Movie S6

Supplementary Movie S7

Supplementary Movie S8

Supplementary Movie S9

Supplementary Movie S10

Supplementary Datasets

## Figures and Tables

**Figure 1:**
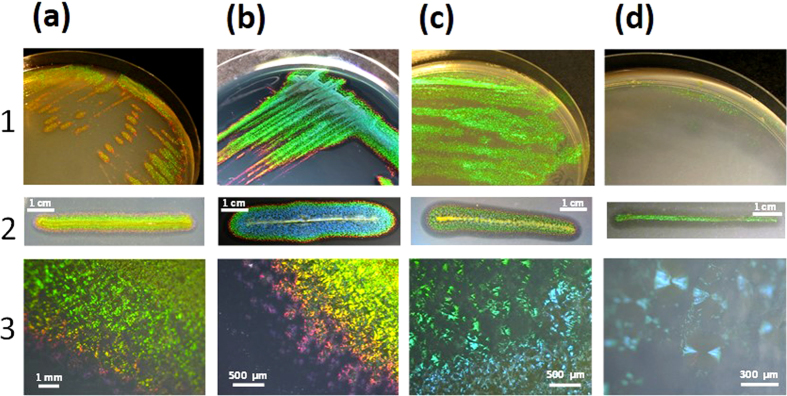
Observations under direct epi-illumination of *Cellulophaga lytica* CECT 8139’s colonies grown on several agar media (see also Supplementary Movies S1-S5). *C. lytica* was aerobically grown for 24 h at 25 °C on (**a**) Marine Agar (MA), (**b**) MA supplemented with black ink (Paper Mate^©^ 1% v/v), (**c**) Cytophaga agar (CYT) or (**d**) Low Nutrient (LN) medium (See *Methods* for media compositions). In (1,2), macroscopic pictures were taken under oblique epi-illumination with a light angle of 67.5°. (1), standard isolation procedure. (2), streaking procedure using a thin 5 cm-linear streak for inoculation^19^. In (3), optical digital microscopy (Keyence©, VHX-1000E) was used and pictures were taken at ×50 (**a**), ×100 (**b**,**c**) or ×200 (**d**) magnifications. To avoid specular reflections, the camera was oriented at a 60° angle from the Petri dish. In macroscopic pictures (1,2), the green iridescence appearance is dominant but blue and other colors (such as red and violet at the colony edges) are also observed. In (**b**), a little ink (1% v/v) was added to the culture medium in order to limit reflections of incident light into the agar at the time of observation. Gliding motility^33^ can be identified as the spreading zone from the colony center (for instance, see 2**(a–c)**). Bacterial agarolysis corresponds to the dark halo visible on colony edges. In the particular LN condition, *C.lytica* forms transparent colonies which appear green iridescent when moving the Petri dish and/or changing the illumination-observation angles (see also Supplementary Movie S3). In microscopic pictures (Keyence© microscopy), the iridescent “speckles” are well visible. Green speckles are dominant but yellow, orange, red, and violet “pointillistic” iridescences are easily observed at the colony edges.
