# Supplementary material for: A unique self-organization of bacterial sub-communities creates iridescence in Cellulophaga lytica colony biofilms
Source: Sci Rep. 2016 Jan 28;6:19906. doi: 10.1038/srep19906 (PMC4730217; doi:10.1038/srep19906)
Supplement: Supplementary Information [file srep19906-s1.pdf]

SUPPLEMENTARY INFORMATION for:

A unique self-organization of bacterial sub-communities creates iridescence in *Cellulophaga lytica* colony biofilms

Betty Kientz[a,#], Stephen Luke[b], Peter Vukusic[b,#], Renaud Péteri[c,#], Cyrille Beaudry[c], Tristan Renault[d], David Simon[c], Tâm Mignot[e] & Eric Rosenfeld[a,#,1]

[a]UMR 7266 CNRS- Littoral Environnement et Sociétés, Microbial Physiology Group - Université de La Rochelle, Avenue Michel Crépeau, 17042 La Rochelle, France

[b]School of Physics, University of Exeter, Exeter EX4 4QL, United Kingdom

[c]Laboratoire Mathématiques, Image et Applications EA 3165, Université de La Rochelle, France.

[d]Institut Français pour la Recherche et l'Exploitation de la Mer, Unité Santé Génétique et Microbiologie des Mollusques, Laboratoire de Génétique et de Pathologie des Mollusques Marins, La Tremblade, France.

[e]UMR 7283 CNRS Laboratoire de Chimie Bactérienne, Institut de Microbiologie de la Méditerranée, University of Aix-Marseille, Marseille, France

[#]Equal contribution of authors.

[1]Correspondence to: eric.rosenfeld@univ-lr.fr

Contents

- **Legends for supplementary movies (S1 to S10)**---------------p. 2-4
- **Dataset S1**--p. 5-9
- **Dataset S2**--p. 10-12
- **(Dataset S3,** uploaded as excel file)
- **Dataset S4**--p. 13-17
- **Dataset S5**--p. 18
- **Dataset S6**--p. 19-22

<u>Movie S1:</u> MA-grown *C. lytica* (x100, plate rotation).

Growth conditions: C. lytica CECT8139 strain was isolated by streaking on a marine agar (MA) plate and incubated 24 h at 25°C. Specifically for this experiment, a little ink (1 % v/v, Paper mate®) was added to the culture medium in order to limit reflections of incident light into the agar at the time of observation.

Observation: Iridescence of *C. lytica* colonies was observed using the Keyence microscope at a x100 magnification by continuously rotating the plate.

<u>Movie S2</u>: CYT-grown *C. lytica* (x200, plate rotation).

Growth conditions: C. lytica CECT8139 strain was isolated by streaking on a cytophaga agar (CYT) plate and incubated 24 h at 25°C.

Observation: Iridescence was observed by using the Keyence microscope at a x200 magnification by continuously rotating the plate.

<u>Movie S3</u>: LN-grown *C. lytica* (x200, plate rotation).

Growth conditions: C. lytica CECT8139 strain was isolated by streaking on a low nutrient agar (LN) plate and incubated 24 h at 25°C.

Observation: Iridescence was observed by using the Keyence microscope at a x200 magnification by continuously rotating the plate. In this condition, colony was transparent and only blue-green iridescence was visualized.

<u>Movie S4</u>: MA-grown *C. lytica* (x100, high to low incidence angles).

Growth conditions: C. lytica CECT8139 strain was isolated by streaking on a marine agar (MA) plate and incubated 24 h at 25°C.

Observation: "Pointillistic" iridescence and transitory colorations of *C. lytica* colonies were analyzed at x100 magnification using the Keyence microscope equipped with a VH-K20 lens

ring (see *Methods*). By moving the VH-K20 lens ring from right to left, positions of illumination were varying continuously from high to low light incidence angles. See also **Fig. 1(a-d)** in the manuscript.

Movie S5: CYT-grown *C. lytica* (x100, high to low incidence angles).

Growth conditions: C. lytica CECT8139 strain was isolated by streaking on a cytophaga agar (CYT) plate and incubated 24 h at 25°C.

Observation: "Pointillistic" iridescence and transitory colorations of *C. lytica* colonies were analyzed at a x100 magnification using the Keyence microscope equipped with a VH-K20 lens ring (see *Methods*). By moving the VH-K20 lens ring from right to left, positions of illumination were varying continuously from high to low light incidence angles.

Movie S6: Gliding motility and cell organization of *C. lytica* CECT 8139 in an iridescent zone of the colony biofilm (CYT agar medium).

Movements were recorded during 40 sec (one photo every three seconds) using time-lapse phase contrast microscopy (100x objective, see *Methods*).

Movie S7: Gliding motility and cell organization of *C. lytica* CECT 8139 in an iridescent zone of the colony biofilm (MA agar medium).

Movements were recorded during 2 x 1 min (one photo every three seconds) using time-lapse phase contrast microscopy (100x objective, see *Methods*).

Movie S8: Gliding motility and cell organization of *C. lytica* CECT 8139 in an iridescent zone of the colony biofilm (LN agar medium).

Movements were recorded during 40 sec (one photo every three seconds) using time-lapse phase contrast microscopy (100x objective, see *Methods*).

<u>Movie S9</u>: Observation of *C. lytica* CECT 8139 cells grown in sNA agar medium (no iridescence in the colony biofilm).

Pictures were recorded during 30 sec (one photo every three seconds) using time-lapse phase contrast microscopy (100x objective, see *Methods*).

<u>Movie S10</u>: Observation of *C. lytica* CIP 103822 cells grown in CYT agar medium (no iridescence in the colony biofilm).

Pictures were recorded during 40 sec (one photo every three seconds) using time-lapse phase contrast microscopy (100x objective, see *Methods*).

Dataset S1. Example of image processing steps for determination of iridescent areas at the edges of a *C. lytica* CECT 8139 colony. Images are those used in Figure 2(a-d) (see Main text). **Panel (A)**, upper zone of the original image; **Panel (B)**, lower zone; **Panel (C)**, original image. **Panels (D)** and **(E)** are tables that summarize the color and luminance variations and the area-proportions of iridescence, respectively.

Optical digital microscopy images (x200) were taken at high (1), intermediate (2) and low (3) light incidence angles. The identified iridescent pixels are shown in white. Color (C) (or luminance, L) maps show the iridescent pixels that vary in color (or luminance) between images 1 and 2 or 2 and 3. Color and luminance (C+L) maps were obtained by merging C maps to L maps. Using our program, we could also obtain intersection maps showing the iridescent pixels that vary in both color and luminance [see examples “Inter 1-2” given at the bottom left in Panels (A,B)].

Threshold wavelength for color variation was 7.5 nm. Luminance and saturation thresholds were 0.15 and 0.25, respectively.

The iridescent speckless which appear/disappear or disappear/appear between two images (two angles) were recorded. Saturation and luminance were computed separately but the data were merged within the same map (see also *Methods*).

In panel (B), the non-colonized area (dark zone) and specular reflections were masked for calculations.

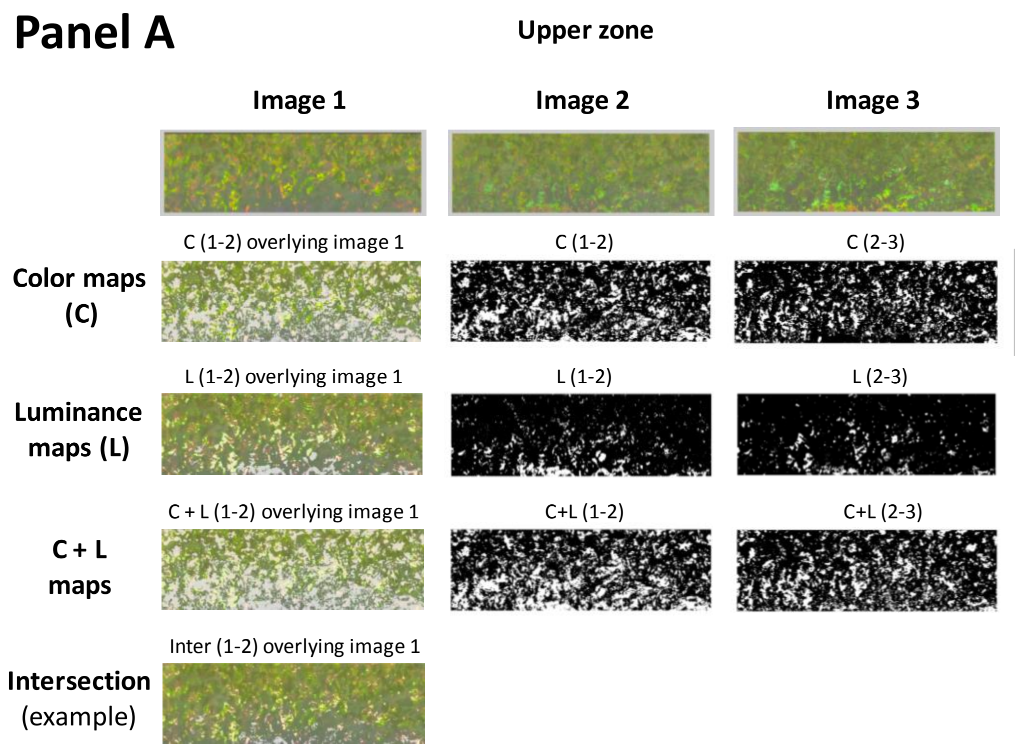
Panel A
Upper zone
Image 1
Image 2
Image 3
C (1-2) overlying image 1
C (1-2)
C (2-3)
Color maps (C)
L (1-2) overlying image 1
L (1-2)
L (2-3)
Luminance maps (L)
C + L (1-2) overlying image 1
C+L (1-2)
C+L (2-3)
C + L maps
Inter (1-2) overlying image 1
Intersection (example)

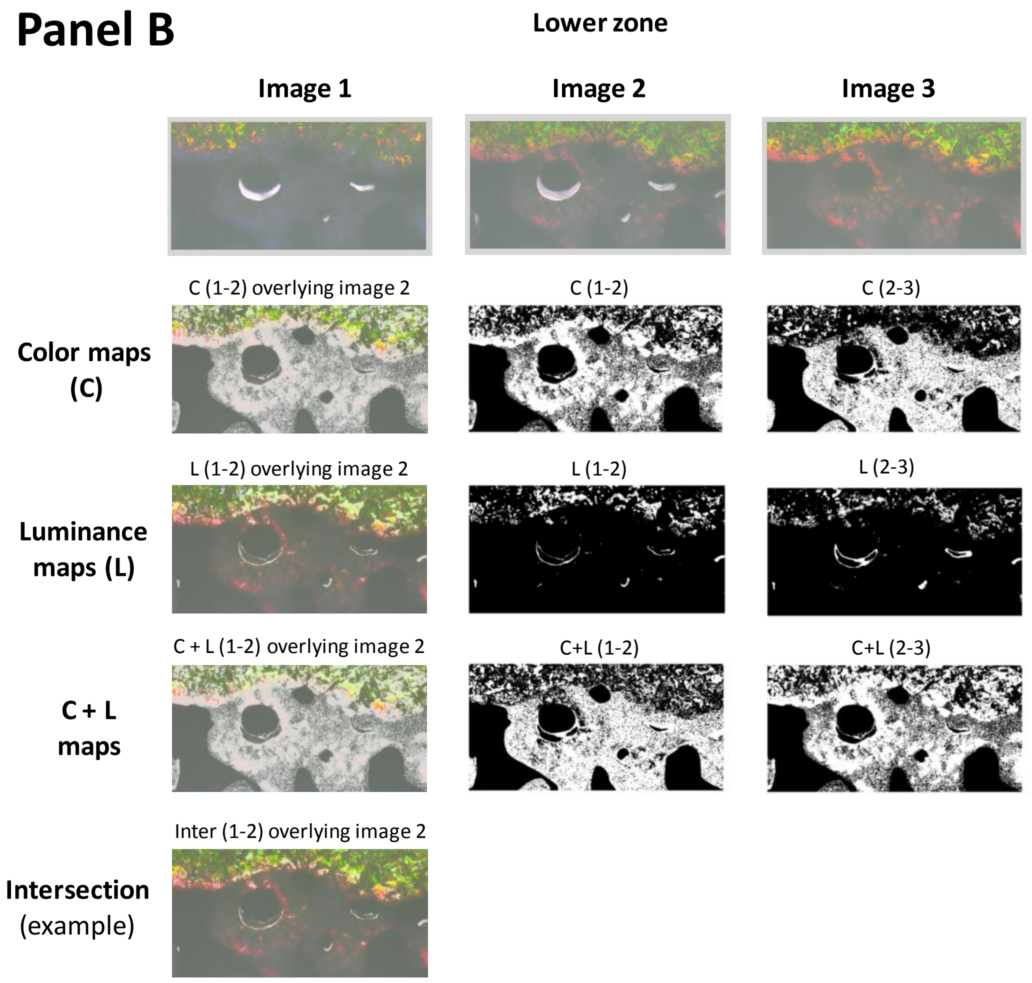
Panel B
Lower zone
Image 1
Image 2
Image 3
C (1-2) overlying image 2
C (1-2)
C (2-3)
Color maps (C)
L (1-2) overlying image 2
L (1-2)
L (2-3)
Luminance maps (L)
C + L (1-2) overlying image 2
C+L (1-2)
C+L (2-3)
C + L maps
Inter (1-2) overlying image 2
Intersection (example)

Panel C

Merged maps

Map of color

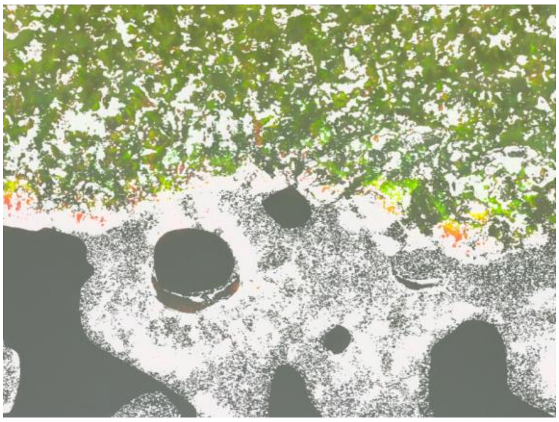

Map of color and luminance

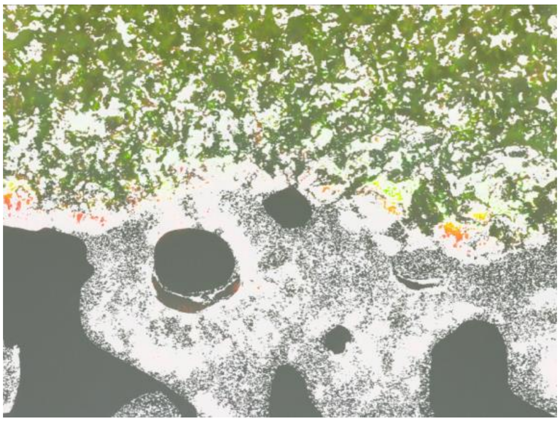

Panel D

Statistical analysis of angle-dependent color and luminance variations at the edges of a *C. lytica* CECT 8139's colony (see also Fig. 2(a,b) in the main text).

**Area calculations**	**µm²**
Total image area	1 843 200
Colony area	1 568 200
Iridescence area	1 071 900
Non-colonized area (dark zone with or not specular reflections)[1]	274 950
Color variation between images 1 & 2	172 690
Luminance variation between images 1 & 2	38 498
Color variation between images 2 & 3	123 340
Luminance variation between images 2 & 3	21 571
Color variation between images 1 & 3	33 707
Luminance variation between images 1 & 3	5 258
Variation in both color and luminance between images 1 & 2	1 873
Variation in both color and luminance between images 2 & 3	4 862
**Percentage calculations**	**%**
Iridescent colony area	68.35
Zones varying in color that also vary in luminance between images 1 & 2	1.1
Zones varying in luminance that also vary in color between images 1 & 2	4.9
Zones varying in color that also vary in luminance between images 2 & 3	3.9
Zones varying in luminance that also vary in color between images 2 & 3	22.5
Zones that have varied in color between images 1 & 2 and that also vary between 2 & 3	19.5
Zones that vary in color between images 2 & 3 and that have also varied between 1 & 2	13.6
Zones that have varied in luminance between images 1 & 2 and that also vary between 2 & 3	27.3
Zones that vary in luminance between images 2 & 3 and that have also varied between 1 & 2	24.3

Images 1, 2 and 3 are those shown in Fig. 2(a).

[1]In the lower zone of the original image, the non-colonized area (dark zone) and specular reflections were masked for calculations.

Panel E

Area-proportions of iridescence at the edges of a *C. lytica* CECT 8139's colony (see also Fig. 2(a-d) in the main text).

		Area of iridescence (10^3 µm^2)	Iridescence *vs* total colony area (%)
**Upper zone**	From image 1 to 2	261.8	34.1
	From image 2 to 3	198.3	25.8
	Total variation in color	297.7	38.8
	Total variation in color and luminance	339.1	44.2
**Lower zone**[1]	From image 1 to 2	609.0	63.8
	From image 2 to 3	595.9	62.5
	Total variation in color	775.8	81.3
	Total variation in color and luminance	807.6	84.7
**Whole image**	Total variation in color and luminance	1072[2]	68.35[2]

[1]In the lower zone image, the non-colonized area (dark zone with or not specular reflections) was masked for calculations.

[2]Values obtained from the raw whole image (see Panel D).

Dataset S2. Examples of image processing for determination of iridescent areas in *C. lytica* CECT 8139's colonies grown on MA (Panel A, a-g) or CYT (Panel B, h-k). Images are those analyzed in Dataset S3.

Optical digital microscopy images were taken at high (1), intermediate (2) and low (3) light incidence angles. Magnifications were x100 for (**a, c, e, h, j**) and x200 for (**b, d, f, g, I, k**), respectively.

The identified iridescent pixels are shown in white on binary (mask) images. Color (C) (or luminance, L) maps show the iridescent pixels that vary in color (or luminance) between images 1 and 2 or 2 and 3. Iridescent pixels that appear or disappear in at least one image were recorded.

Color and luminance (C+L) maps are not shown.

Short description:

Images (**a, c, e**) are examples containing specular reflections.

In (**a, e, f**), the extreme edges of the colony are visible.

In (**d**), an islet-like region of the MA-grown colony was analyzed.

On CYT agar medium (**h-k**), islet-like regions were often observed at the extreme edges of the colonies. Blue iridescence was more visible on this medium.

Panel A

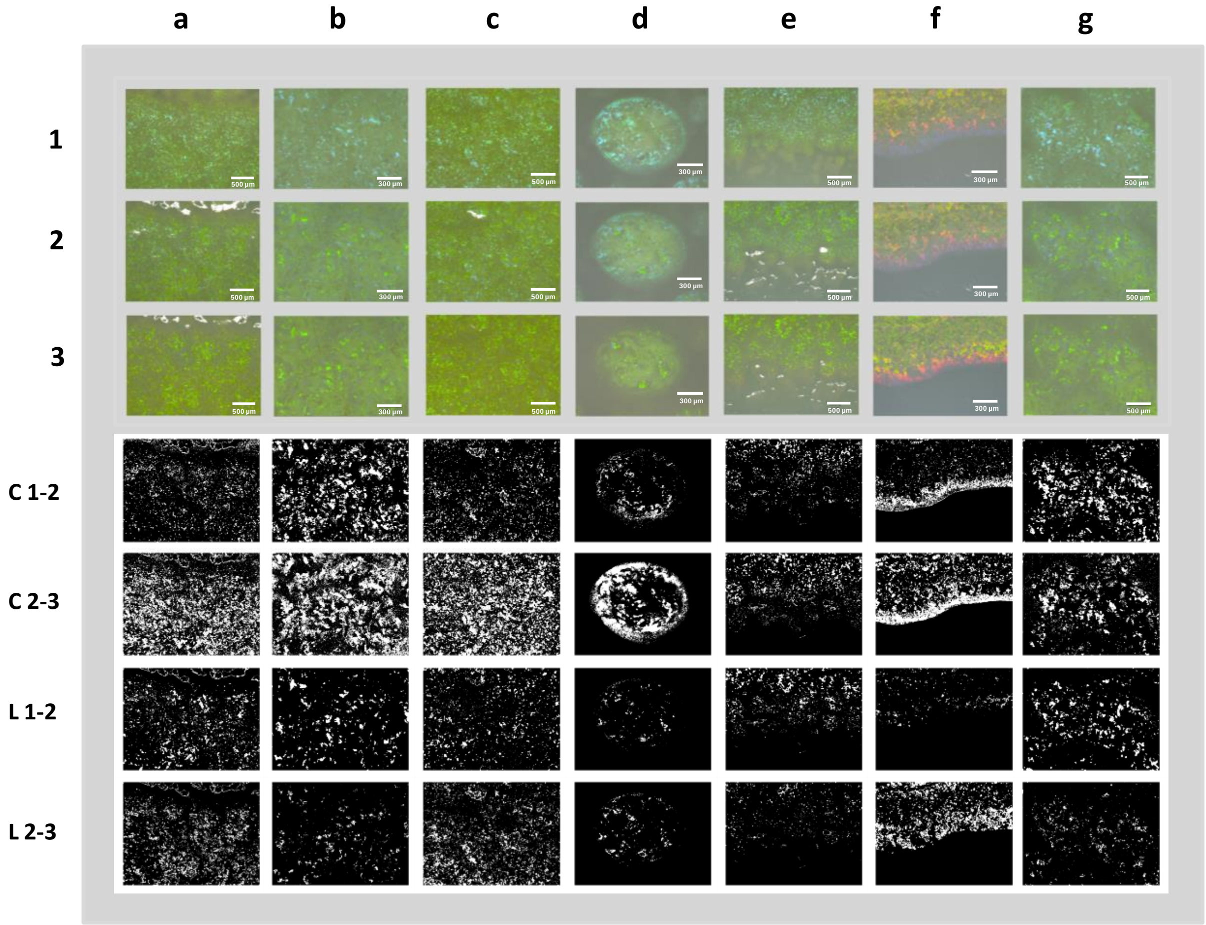

Panel B

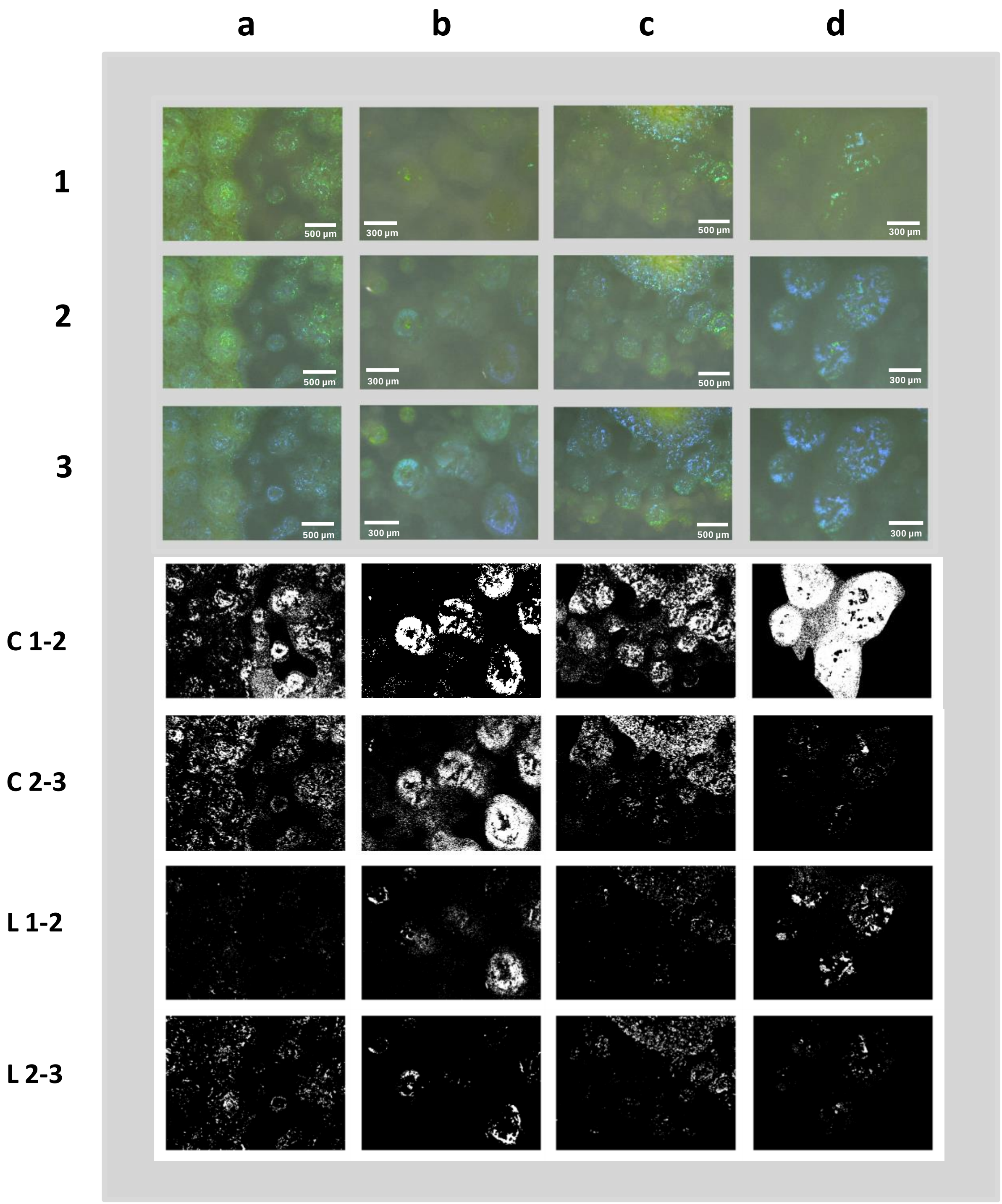

Dataset S3 (uploaded as Excel file). **Statistical analyses of angle-dependent color and luminance variations in *C. lytica* CECT 8139 colonies.** See also Dataset S2.

Dataset S4 (related to Figure 2(f)). **Examples of determinations of iridescence elemental unit sizes in *C. lytica* CECT 8139's colonies grown on MA (A-B) or CYT (C-D).**

Direct observations of colored colonies were performed under epi-illumination by using a numeric Keyence microscope (VHX-1000E). A VHX-1100 camera with a VH-Z20R/Z20W objective lens was adjusted at x100 (**A,C**) or x200 (**B,D**) magnifications. To avoid specular reflections, the VH-S30 supporting mount of the camera was oriented at a 60° angle from the plate. The DEPTH UP/3D tool corresponding to the depth-from-defocus (DFD) process was employed to focus on all optical fields and to improve image quality.

Size of the iridescent "speckles" were determined using a specific image processing program (see *Methods*). The examples **C-D** (CYT-grown colony) are enlarged images of those shown in **Figure 2(f)** (See Main text) and contain additional computational results.

Panel A

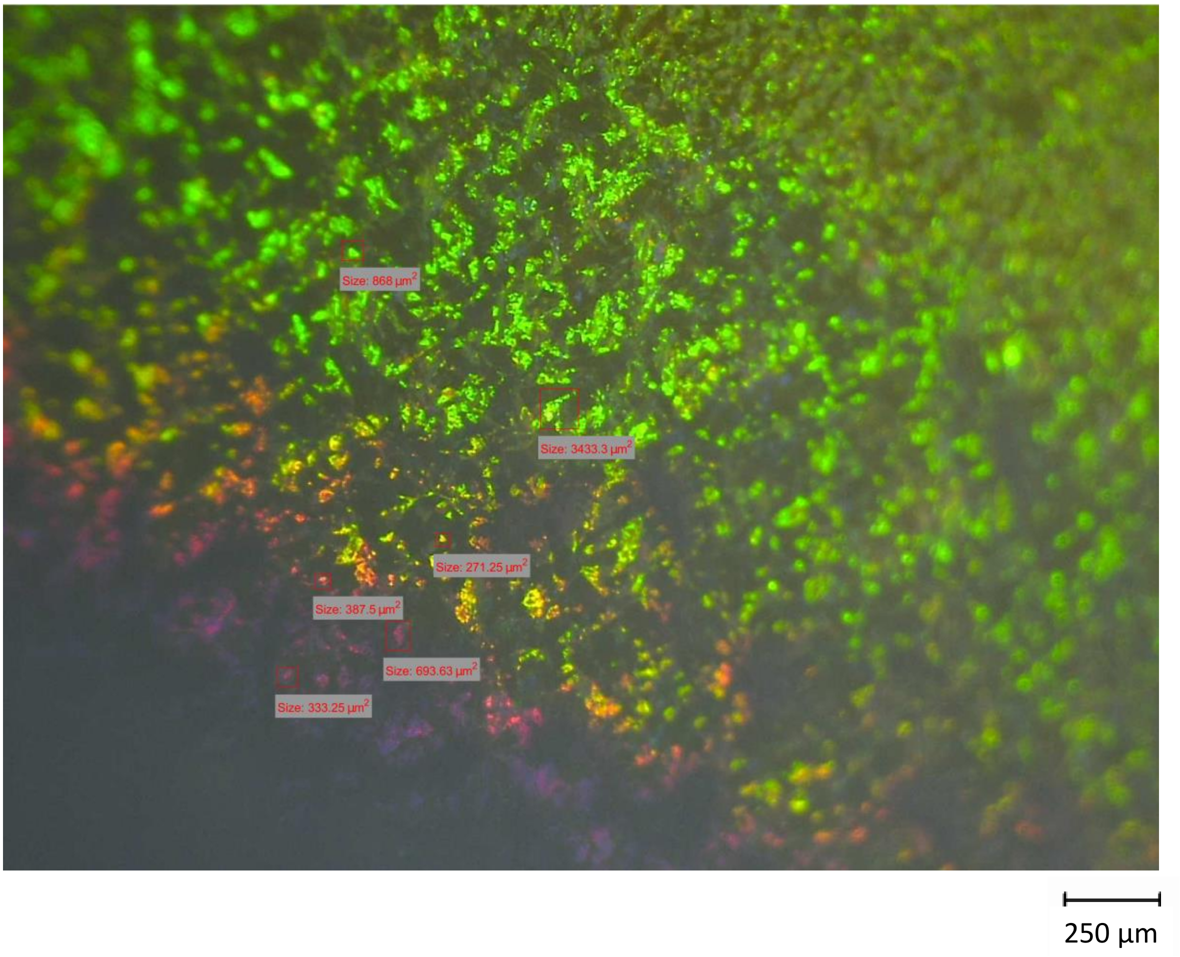

Size: 868 µm²
Size: 3433.3 µm²
Size: 271.25 µm²
Size: 387.5 µm²
Size: 693.63 µm²
Size: 333.25 µm²
250 µm

Panel B

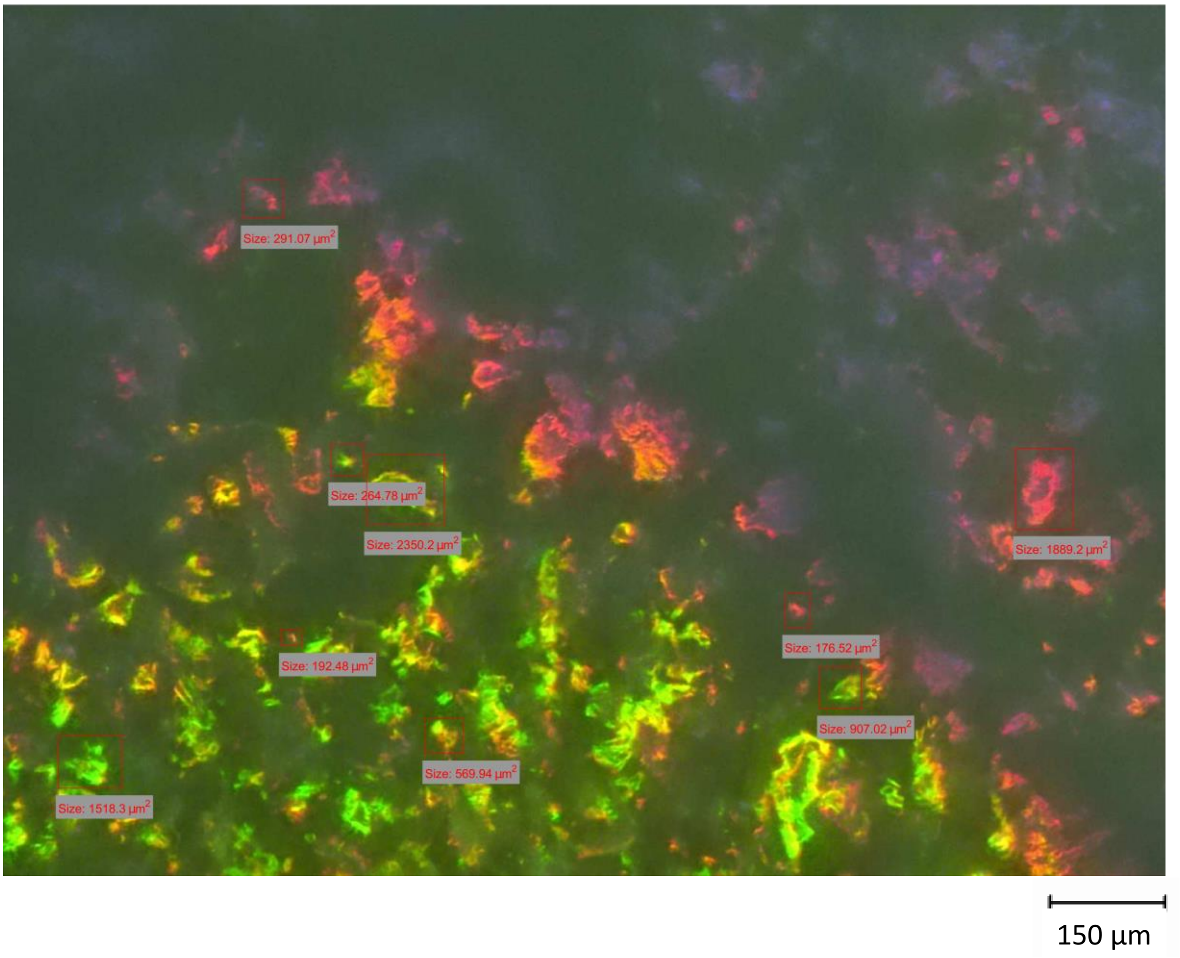
Size: 291.07 μm^2
Size: 264.78 μm^2
Size: 2350.2 μm^2
Size: 1889.2 μm^2
Size: 176.52 μm^2
Size: 192.48 μm^2
Size: 907.02 μm^2
Size: 569.94 μm^2
Size: 1518.3 μm^2
150 µm

Panel C

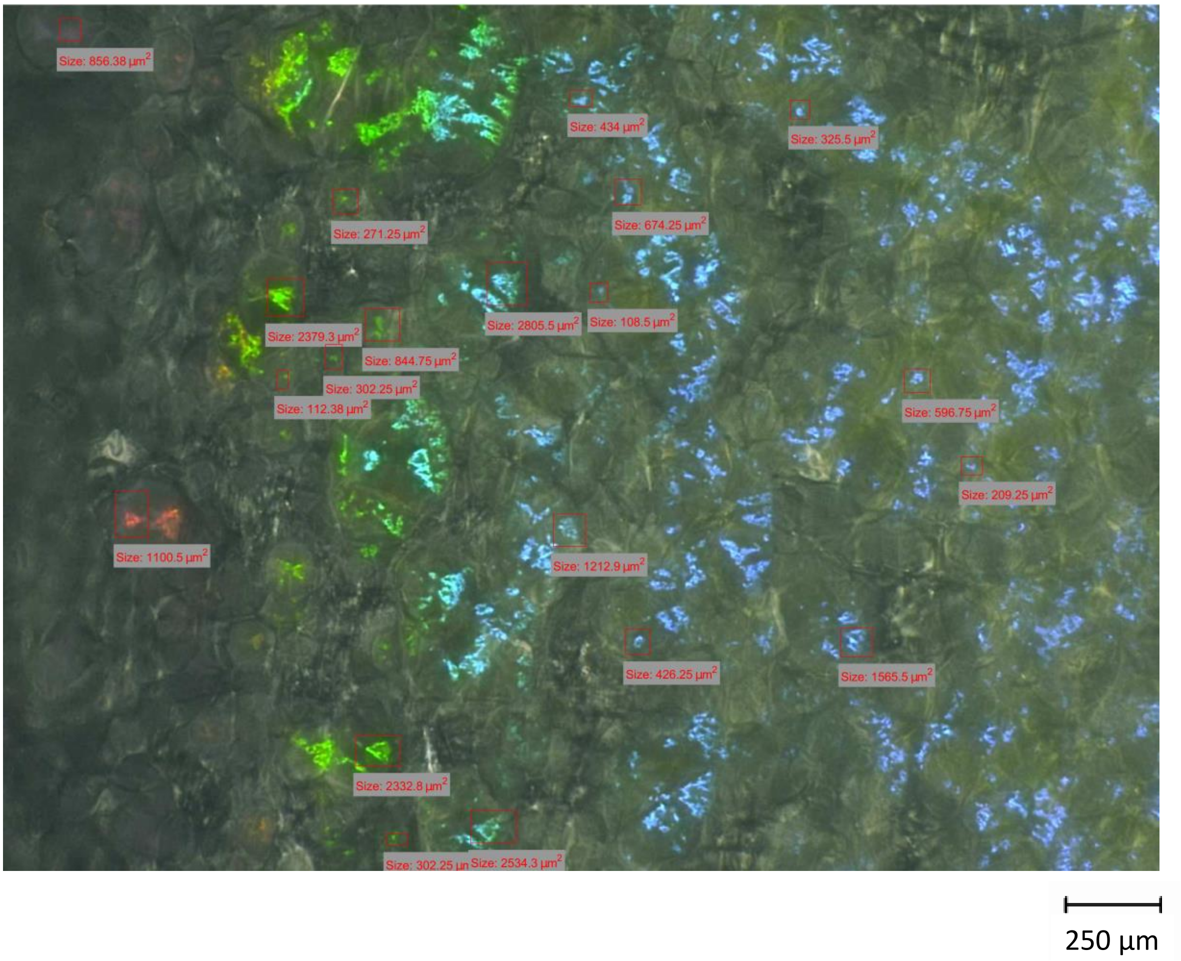
Size: 856.38 µm²
Size: 434 µm²
Size: 325.5 µm²
Size: 271.25 µm²
Size: 674.25 µm²
Size: 2379.3 µm²
Size: 844.75 µm²
Size: 2805.5 µm²
Size: 108.5 µm²
Size: 302.25 µm²
Size: 112.38 µm²
Size: 596.75 µm²
Size: 209.25 µm²
Size: 1100.5 µm²
Size: 1212.9 µm²
Size: 426.25 µm²
Size: 1565.5 µm²
Size: 2332.8 µm²
Size: 302.25 µn
Size: 2534.3 µm²
250 µm

Panel D

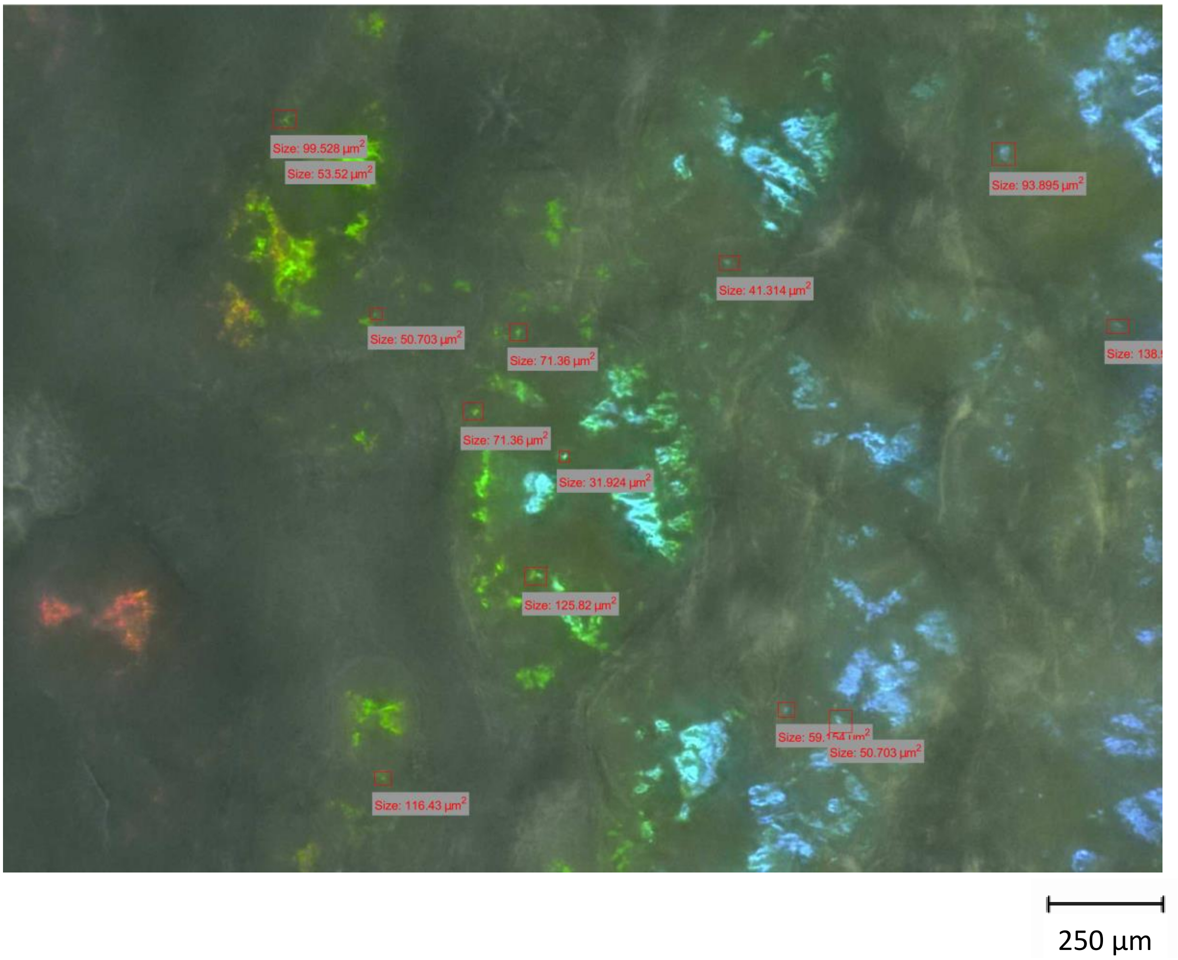

Size: 99.528 µm2
Size: 53.52 µm2
Size: 93.895 µm2
Size: 41.314 µm2
Size: 50.703 µm2
Size: 71.36 µm2
Size: 71.36 µm2
Size: 31.924 µm2
Size: 125.82 µm2
Size: 50.703 µm2
Size: 116.43 µm2
250 µm

Dataset S5 (related to Figure 4). **Examples of TEM cross-section images of *C. lytica* colonies grown under iridescent (A,B) or non iridescent (C,D) conditions**. Iridescent conditions were: *C. lytica* CECT 8139 grown on CYT (**A**) or LN (**B**) media. Non iridescent conditions were: *C.lytica* CECT 8139 grown on salted Nutrient Agar (sNA) (**C**) or *C. lytica* CIP103822 grown on CYT (**D**).

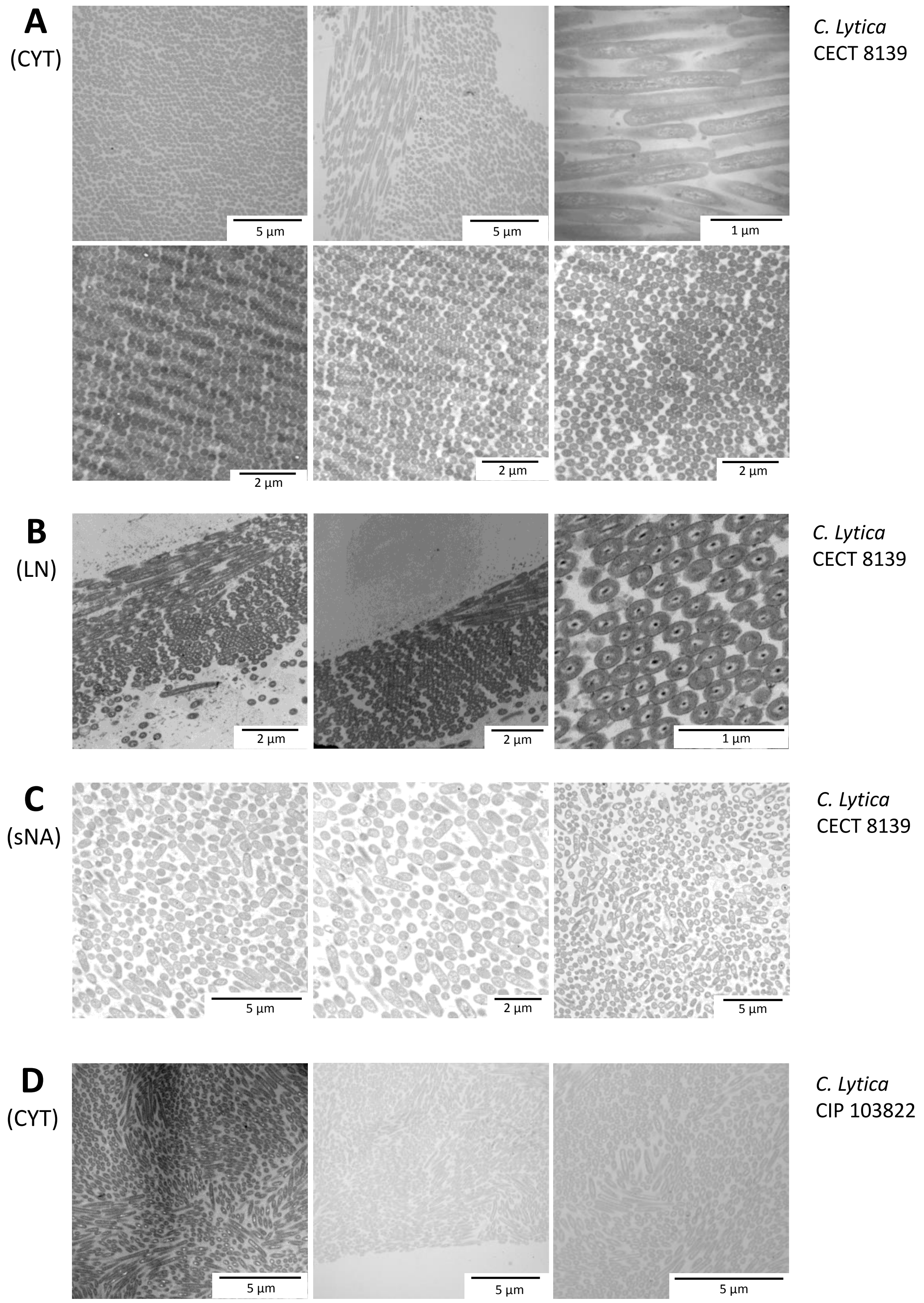

Dataset S6 (related to Figure 3(d,e)). **Mathematical morphology analysis of TEM images.** Positive samples are presented in **Panel 1** (CECT 8139 strain on CYT) and negative ones in **Panel 2** (CIP103822 strain on CYT or CECT 8139 strain on sNA). **Panel 3** is a table that summarizes the statistics of the structures (number of extracted cells, typical value of the cells' diameter, mean distances). **Panel 4** is a scheme showing the principle of repetition of hexagonal cell patches in iridescent colony biofilms.

In **Panels 1-2**, each figure shows:

– The original TEM image

– The extracted cells and the associated Delaunay graph

– The frequency plot of the mean distance from the 6 nearest-neighbours of each cell.

The Delaunay graphs show that the spatial arrangement of the positive samples are visually more regular than the negative ones; there are some strong 'local' deformations on the Delaunay graphs of negative samples. This observation tends to be confirmed by the standard deviations on the 6 nearest neighbours (a measure more robust than the nearest neighbour), and by the spread of the histograms in the case of negative samples (e.g. sNA condition). Moreover, the mean and standard deviation on the positive samples are quite similar on the different images.

Panel 1

C. *lytica* CECT 8139 grown on CYT

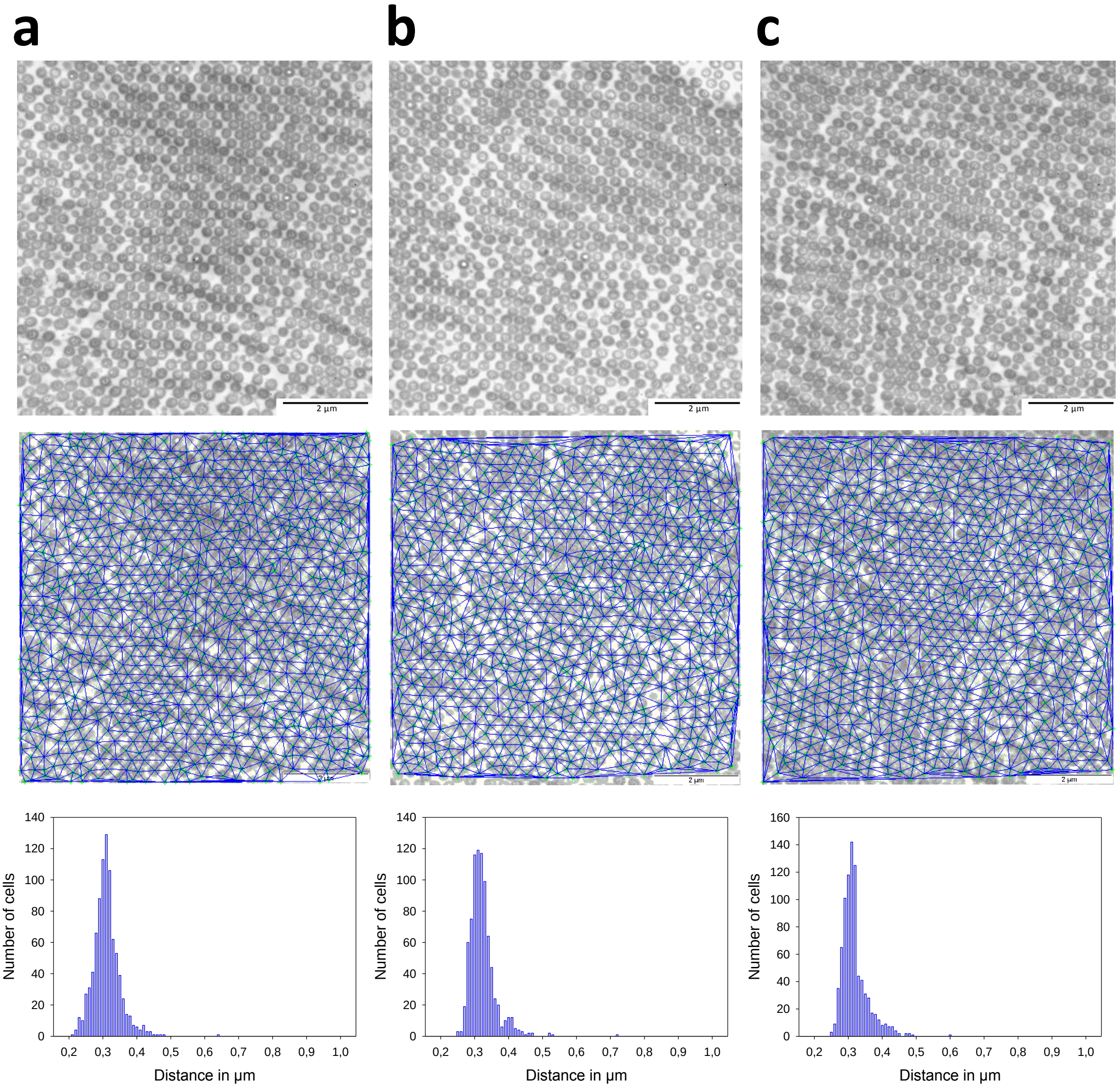

Panel 2

C. lytica CIP103822 grown on CYT

C. lytica CECT 8139 grown on sNA

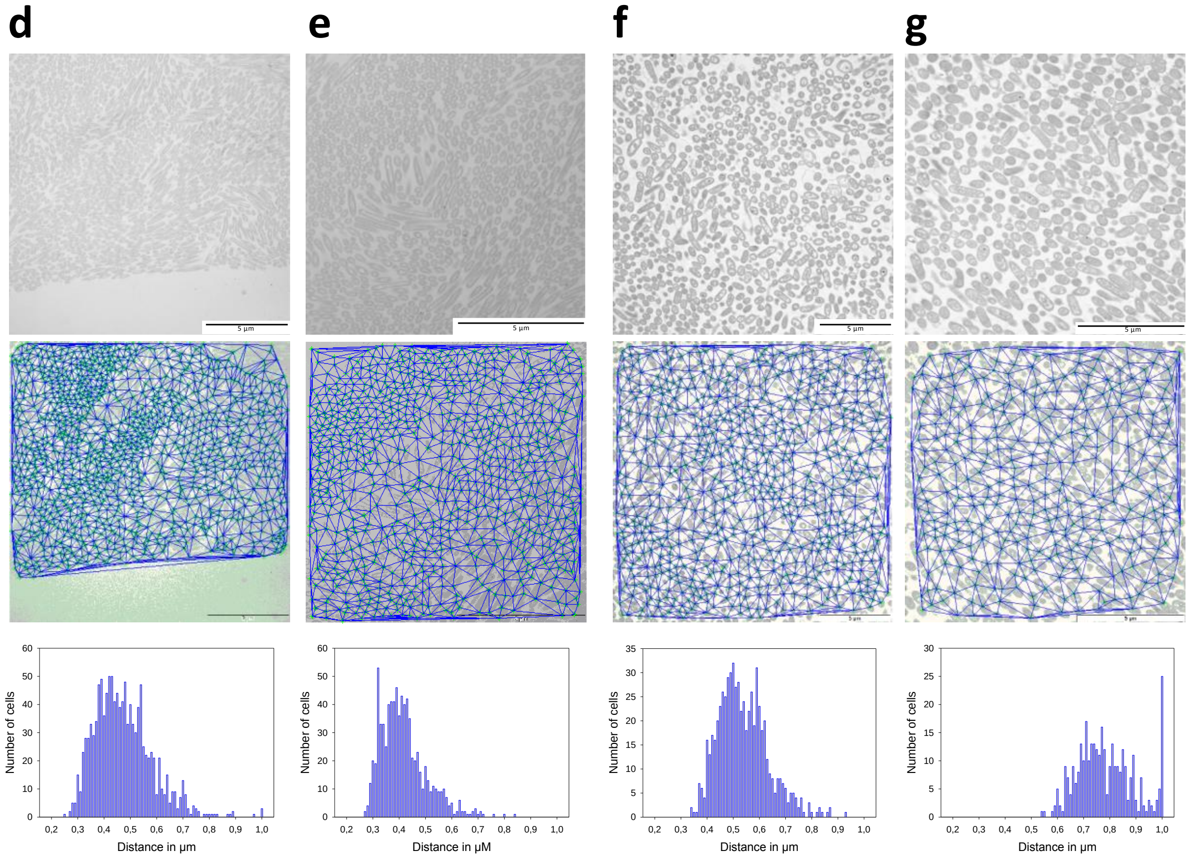

Panel 3

TEM images	Iridescent conditions<br>*C. lytica* CECT 8139 on CYT medium<br>a	 <br>b	 <br>c	Non-iridescent conditions<br>*C. lytica* CIP 103822 on CYT medium<br>d	 <br>e	 *C. lytica* CECT 8139 on sNA medium<br>f	 <br>g
**Extracted parameters**							
Number of extracted cells	830	868	824	1178	787	645	330
Typical value of the cells' diameter (in µm)	0.277	0.285	0.285	ND	ND	ND	ND
Mean distance from the 6 nearest neighbours (6 NN) (µm)	**0.318**	**0.311**	**0.322**	**0.474**	**0.416**	**0.538**	**0.795**
Standard deviation of the mean distance from the 6 NN	**0.038**	**0.039**	**0.038**	**0.113**	**0.087**	**0.096**	**0.128**
Mean distance from the nearest neighbours (µm)	0.241	0.223	0.233	0.305	0.281	0.358	0.508
Standard deviation of the mean distance from the NN (µm)	0.031	0.041	0.033	0.083	0.061	0.083	0.106

Panel 4

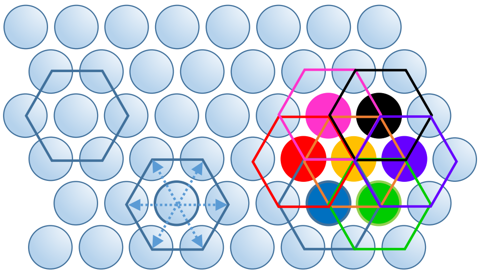

Principle of repetition of hexagonal cell patches in iridescent colony biofilms (polycrystalline nature).
The periodicity was quantified by the associated mean distances from the six nearest neighbours and standard deviations (Panel 1,3).
